# Modeling the Effect of Treatments on Prostate Cancer-Specific Mortality and the Relevant Geographical Variation and Racial Disparities

**DOI:** 10.3390/cancers18060983

**Published:** 2026-03-18

**Authors:** Wensheng Zhang, Christopher Williams, Guangdi Wang, Kun Zhang

**Affiliations:** 1 Bioinformatics Core of Xavier RCMI Center of Cancer Research, Xavier University of Louisiana, New Orleans, LA 70125, USA; 2 Department of Computer Science, Xavier University of Louisiana, New Orleans, LA 70125, USA; 3 School of Pharmacy, Xavier University of Louisiana, New Orleans, LA 70125, USA; cwilli35@xula.edu; 4 Department of Chemistry, Xavier University of Louisiana, New Orleans, LA 70125, USA; gwang@xula.edu

**Keywords:** prostate cancer, cancer-specific mortality, treatment, disparity, geographical variation

## Abstract

**Background/Objectives**: African American (Black) prostate cancer (PCa) patients have a higher risk of dying from the disease and are less likely to undergo radical treatment than European Americans (White). The disparities in PCa-specific mortality (PCSM) and mortality rate (PCSMR) vary geographically. This study investigated the impact of treatments on PCSM, PCSMR and the relevant disparities. **Methods**: Using the Cox PH model and other statistical methods, we analyzed two datasets extracted from the SEER and PLCO databases. The SEER dataset contains 637,093 White patients and 110,839 Black patients. The PLCO dataset included 7463 Whites and 495 Blacks, and supplemented the SEER data with information on PCa family history (pros_fh). **Results**: Analysis of SEER data showed that the relative mortality risk (RR) of patients undergoing surgery alone was significantly lower than that of patients receiving radiotherapy alone or a combination of surgery and radiotherapy. Black patients’ RR estimated by the model including treatment was substantially smaller than that estimated by the reduced model excluding treatment. The differences between Black and White in the three-nine-year PCSMR of patients with high-grade or non-localized cancer were significantly correlated with the differences in surgery alone rate (r < −0.65, *p* < 0.001). Regression-based mediation analysis indicated that treatment disparity had a significant direct effect on mortality disparity and did not mediate the effect of age disparity. Analysis of PLCO data showed that pros_fh had no significant effect on survival but confirmed the survival advantage of surgery over radiotherapy. **Conclusions**: The results of this study support the hypothesis that, for PCa patients in the United States, geographical variation in treatment disparity partially explains variation in mortality disparity.

## Simple Summary

Black American prostate cancer (PCa) patients have a higher risk of dying from the disease and are less likely to undergo radical treatment than White Americans. Mortality disparities vary geographically. This study investigated the impact of treatments on PCa-specific mortality and the relevant disparities. Using the Cox proportional hazards (Cox PH) model and other statistical methods, we analyzed two datasets extracted from the SEER and PLCO databases. The primary findings were as follows: the relative mortality risk (RR) of patients undergoing surgery alone was significantly lower than that of patients receiving radiotherapy alone or a combination of surgery and radiotherapy; Black patients’ RR estimated by the model including treatment was substantially smaller than that estimated by the reduced model excluding treatment; and across geographic areas, racial disparities in mortality were associated with disparities in treatment. These findings provide new insight into the etiology of the poorer survival of Black patients.

## 1. Introduction

A major racial disparity in prostate cancer (PCa) in the United States is that African American (Black) patients have a greater risk of dying from the disease than European American (White) patients [1]. While numerous molecular differences in PCa tissue among races have been identified [2,3], strong evidence for an etiological relationship between them and mortality disparity is rare, if not lacking. For example, the fraction of tumors with TMPRSS2-ERG fusion in White patients is approximately 2 times the fraction in Black patients, but the association between the survival profile of PCa patients and this genomic alteration is not significant, as reported in most of the relevant studies [4,5,6,7]. Empirical analysis of the observational data collected by large-scale epidemiology projects and biomedical information systems is still the primary approach for dissecting the mortality disparity.

Based on the autopsy results of patients from the Detroit registry of The Surveillance, Epidemiology and End Results (SEER) project, Powell et al. proposed that prostate cancer grows more rapidly in Black men than in White men and/or that earlier transformation from latent to aggressive prostate cancer occurs in Black men than in White men [8]. However, the plausibility of this conception in explaining PCa mortality disparity is compromised by the results of multiple relevant studies. An observational cohort analysis of 1270 veterans diagnosed with prostate cancer and followed for 11 to 16 years at nine medical centers within the Veterans Health Administration implicated that PCa-specific mortality (PCSM) among Black and White patients was similar in equal-access health care systems [9]. An analysis of all data from the American College of Surgeons National Cancer Database (NCDB) of prostate cancer diagnosed between 2004 and 2013 demonstrated that White men had superior overall survival (OS) compared with Black men in the favorable risk cohort, but race was not a significant prognostic factor for OS in the unfavorable risk cohort [10]. Our previous analysis of SEER nationwide records during 2009–2011 demonstrated that racial disparity in the probability of dying from prostate cancer in the first 6 years after initial diagnosis varies geographically. In particular, for patients with cancers in the same grade category (i.e., high or low grade), the survival stratification between Black and White patients was not significant in approximately half of the geographical areas (i.e., counties or parishes) [11]. This finding suggests that “equal grade, equal outcomes” for Black and White patients is not only a verifiable hypothesis but also an achievable public health goal. A major obstacle to achieving this goal may be the between-race inequality in treatment [12,13]. A recent publication showed that ~85% of the treatment inequity in PCa patients between White and Black may be attributable to a potentially broad range of healthcare delivery factors [14].

In this study, we investigate the impact of treatment types on mortality risk due to prostate cancer and the relevant differences between races by a set of statistical analyses of two datasets extracted from the SEER and the Prostate, Lung, Colorectal and Ovarian (PLCO) databases [15]. Our primary aim is to test the hypothesis that geographical variation in the disparity in PCa-specific mortality rate (PCSMR) can be, at least partially, explained by the variation in treatment disparity. The rationales underlying this hypothesis are that inappropriate medication, including inadequate or delayed treatment and overtreatment, can increase the risk of dying from the disease [16,17,18]; Black patients are less likely to undergo radical treatment than White patients due to some race-related social, economic and cultural factors that affect treatment accessibility and refusal [19,20], and the inequality between races in treatment may vary across communities.

Theoretically, a neighborhood community defined by a county or a 3-digit ZIP code area would be the most appropriate proxy for a geographical unit in this study, because residents within the same community are more likely than those in different communities to share similar socioeconomic and environmental conditions, as well as access to medical services and healthcare options [21]. However, residence information for patients in the SEER database is currently not publicly available. Therefore, we used combinations of Rural–Urban Continuum Codes and states as geographical units. Although these units are not perfect proxies for neighborhood communities, some closely resemble them. For example, the area defined by Louisiana and counties in metropolitan areas with populations greater than one million largely corresponds to the New Orleans metropolitan area.

## 2. Material and Methods

### 2.1. SEER Dataset

SEER collected data according to the relevant regulations and protocols approved by the National Cancer Institute, USA. Consent was obtained from all subjects and/or their legal guardian(s). Active follow-up on cancer patients was performed from diagnosis until death, ascertaining critical information on mortality and survival over time [22].

A working dataset containing 637,093 White and 110,839 Black prostate cancer patients diagnosed during 2000–2017 was extracted from the SEER (RRID:SCR_015499) databases (accessed on 19 September 2022) using SEER*Stat 8.4.0.1 software and our lab-owned R codes. In total, 60,322 PCa-specific deaths occurred during the following periods after initial diagnosis, which had an average length of 105 months. The 747,932 cancer cases were registered in 11 registries with the following distribution: California (286,427), Connecticut (40,958), Georgia (95,566), Hawaii (3986), Iowa (33,782), Kentucky (40,068), Louisiana (54,437), New Jersey (103,081), New Mexico (17,815), Seattle (47,804), and Utah (24,008). The dataset did not include those with missing information on the following variables: “Rural Urban Continuum Codes”, “Median household income inflation adj to 2019”, “Grade through 2017”, “Summary stage 2000 (1998) 2017”, “SEER cause specific death classification”, “Reason no cancer directed surgery” and “Radiotherapy recode”. Based on the original variables, four categorical variables were derived or redefined and were considered in the relevant statistical analysis.

#### 2.1.1. Grade Cluster

Cancer cases were partitioned into low (L) and high (H) grade clusters. The cases in the L cluster were diagnosed as “Well differentiated; Grade I” or “Moderately differentiated; Grade II”. The cases in the H cluster were diagnosed as “Poorly differentiated; Grade III” or “Undifferentiated; anaplastic; Grade IV”. According to the SEER Program Coding and Staging Manual 2012 [23], the cancers coded with grade I, II and III have Gleason Scores (GS) ranking from 2 to 4, 5 to 6 and 7 to 10, respectively (GS corresponding to the code IV is missed in the Manual but it should be over 8) [24]. 

#### 2.1.2. Metro.rac

Patients were partitioned into eight groups based on self-reported races and “Rural Urban Continuum Codes” (termed Metro), i.e., M1 White, M1 Black, M2 White, M2 Black, M3 White, M3 Black, M4 White, and M4 Black. M1, M2, M3 and M4 denote “counties in metropolitan areas gt 1 million pop”, “counties in metropolitan areas of 250,000 to 1 million pop”, “counties in metropolitan areas of lt 250 thousand pop”, and “nonmetropolitan counties”, respectively.

#### 2.1.3. Treatment

Patients were partitioned into four classes, i.e., “Rad−,Surg−”, “Rad−,Surg+”, “Rad+,Surg−” and “Rad+,Surg+”. Surg+ and Surg− denote whether they certainly underwent radical surgery. Rad+ and Rad− denote whether they certainly underwent radiotherapy.

#### 2.1.4. Income

With cutoffs of $50,000 and $70,000 in county-level median annual household income, patients were partitioned into L, M and H classes.

Geographical areas (GARs), such as “counties in metropolitan areas gt 1 million pop, Georgia”, were defined by the combinations of Rural–Urban Continuum Codes and States. They are less specific than the regions defined by specific counties or post codes, which are lacking in the currently available versions of SEER data. GARs were used in analyzing geographical variation and disparity in PCSMR.

### 2.2. PLCO Dataset

The Prostate, Lung, Colorectal, and Ovarian (PLCO) Cancer Screening Trial was a randomized controlled trial. PLCO carried out the trial according to the relevant regulations and protocols approved by the National Cancer Institute, USA. Consent was obtained from all subjects and/or their legal guardian(s).

The dataset was extracted from PLCO database updated in November 2018 (accessed on 24 August 2019). It contained the information of 7463 White and 495 Black prostate cases with complete information on family history of prostate cancer (pros_fh), cancer grades and cancer stage. In total, 436 PCa-specific deaths occurred during the follow-up periods after initial diagnosis, which had an average length of 126 months. In addition to pros_fh, the curative hormone therapy indicator was another variable that was contained in the PLCO dataset but was missing in the SEER data. To use this information, we separated the patients who did not undergo surgery and radiotherapy but took hormone therapy from the “Surg,Rad−” class to form another therapy class termed “Trt−,Horm+”.

The two datasets are summarized in Table 1, focusing on the patient traits, cancer characteristics, and communication attributes related to the variables that were considered in statistical analysis.

### 2.3. Statistical Analysis

Two Cox PH regression models (Model-1 and Model-2), as well as the Kaplan–Meier method, were used to perform survival analysis (with deaths due to prostate cancer as events) on the SEER dataset. The explanatory variables in Model-1 included age, grade.clust, (summary) cancer stage, metro.rac and income. Model-2 was an expanded version of Model-1, with treatment as the additional explanatory variable. The regression model (Model-3) for the PLCO dataset included age, race, pros_fh, grade.clust, cancer stage (AJCC 5th Edition), prostate-specific antigen (PSA) level closest to cancer diagnosis and treatment as the explanatory variables. To improve understanding of analysis results, PSA values were discretized into four integer levels, i.e., 1, 2, 3, and 4, with the three quartiles, where they were 4.6, 6.1 and 8.9, respectively. Missing values were replaced with 1. 

Treatment disparity (disparity in treatment rate) and mortality disparity (disparity in death rate) were quantified using the SEER dataset. Treatment disparity was calculated as the difference between Black and White patients in the proportion of patients who underwent a specific treatment such as surgery alone. PCSMR of a patient group during a specific period after cancer diagnosis was calculated with 1 minus survival probability, which was estimated by fitting a Kaplan–Meier curve. The Pearson correlation coefficient and T-test were used to evaluate the association between geographical area-specific treatment disparities and mortality disparities. Using the E-value method [25], a sensitivity analysis was conducted to estimate the minimum strength of association that an unmeasured confounder would need to fully explain the observed association between treatment and survival. Regression-based mediation analysis [26] was performed to examine the direct effects of treatment disparity and age disparity on mortality disparity, as well as the potential mediating relationship between them.

### 2.4. Software Application

All analyses and graphics were completed by using the relevant functions in the R packages, including “stats (v. 4.5.2)”, “survival (v 3.8.3)” and “grid grid (v. 4.5.2)”, “ggplot2 (v. 4.0.1”, “survminer (v. 0.5.1)”, “EValue(v.4.1.4) ” and “mediation (v. 4.5.1)” as well as our lab-owned R codes.

## 3. Results

### 3.1. Impact of Treatments on Racial Disparity in PCSM

This subsection and the next subsection report the results from the analysis of the SEER dataset.

**Figure 1:**
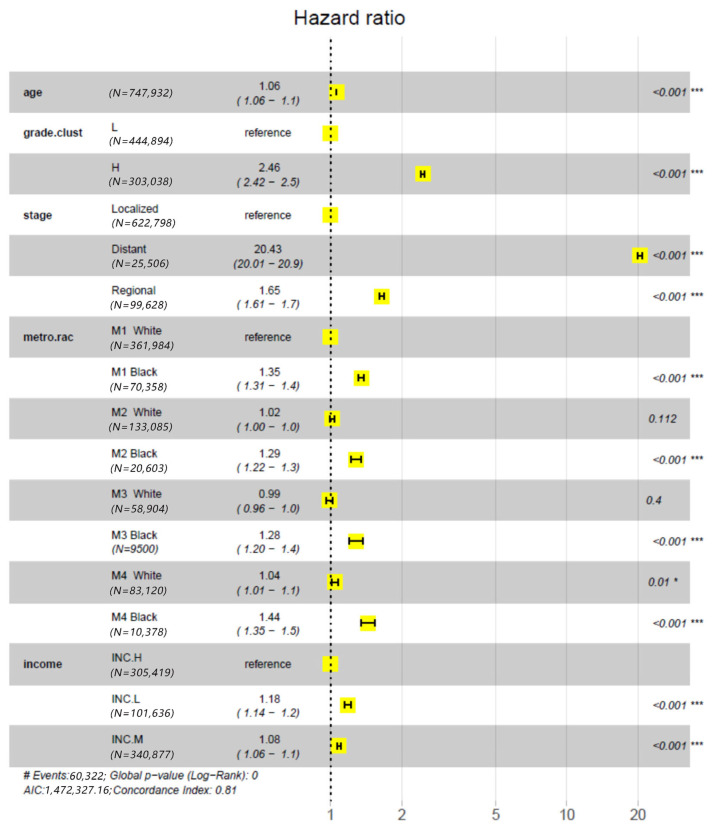
Analysis of racial disparities in prostate cancer-specific mortality (or survival) using Model M-1, which does not include an explanatory variable for treatments. The explanatory variables in the model include age, grade.clust (grade cluster), stage, metro.rac, and (county-level median annual household) income. For each explanatory variable or categorical factor, the center of the filled yellow box represents the point estimate of the Hazard Ratio (HR) and the black “**|─|**” shape represents the 95% CI of the HR estimate. The * and *** indicate *p* < 0.05 and 0.001, respectively.

**Figure 2:**
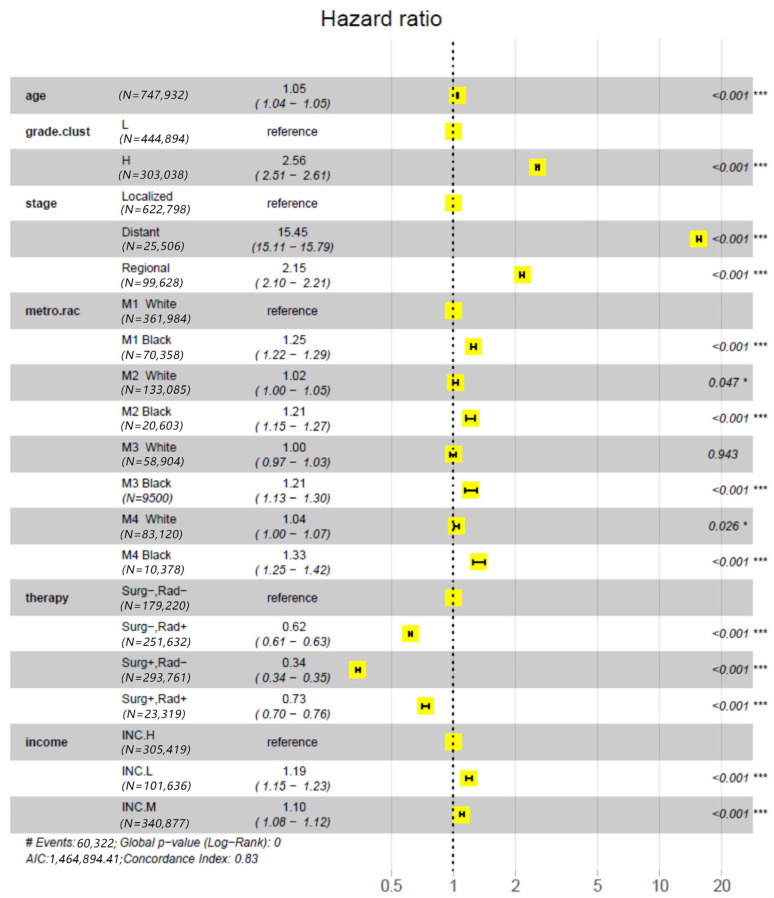
Analysis of racial disparities in prostate cancer-specific mortality (or survival) using Model M-2, which includes an explanatory variable for treatments. Besides treatment (therapy), the explanatory variables in the model include age, grade.clust (grade cluster), stage, metro.rac, and (county-level median annual household) income. For each explanatory variable or categorical factor, the center of the filled yellow box represents the point estimate of the Hazard Ratio (HR) and the black “**|─|**” shape represents the 95% CI of the HR estimate. The * and *** indicate *p* < 0.05 and 0.001, respectively.

The impact of treatments on racial disparity in PCSM was assessed by comparing the results of two Cox PH models, i.e., Model-1 and Model-2. This approach is somewhat similar to the decomposition methods mentioned in the reference [27], which estimate the reduction in disparity in the outcome variable after a causal explanatory variable is accounted for. As mentioned in the method section, the only difference between the two models was that the latter included an explanatory variable for treatments, but the former did not. The effect of each explanatory variable on PCSM was measured by the relative mortality risk (RR, estimated with hazard ratio) of a test group to the baseline (reference) group, RR 95% confidence interval (95% CI) and Wald test *p* value. In particular, we set the M1 White as the baseline group for the variable metro.rac and “Surg−,Rad−” as the baseline group for treatments. The results from the Model-1 analysis (Figure 1) demonstrated that the RR 95% CI substantially deviated from 1.0 in groups M1 Black (1.31–1.39), M2 Black (1.22–1.35), M3 Black (1.2–1.38) and M4 Black (1.35–1.1.53) but not in M2 White (1.0–1.04), M3 White (0.96–1.02) and M4 White (1.01–1.07). Due to the independence of RR from Rural Urban continuum codes in Whites, the RR-1 values of Black groups could be considered as the estimates of mortality disparity. The disparities were obviously compromised when treatments were considered (Figure 2). That is, the RR-1 values calculated from the results of the Model-2 analysis for M1 Black, M2 Black, M3 Black, and M4 Black were 71–75% of those calculated from the results of the Model-1 analysis. This indicates that 25–29% of racial disparity in mortality was accounted for by treatments. In summary, these results demonstrated that controlling for treatment reduced disparities across Rural–Urban Continuum Code strata but did not eliminate them; Black patients still had higher hazard ratios in all strata compared with their White counterparts.

To assess the potential impact of unmeasured confounders on the estimated treatment effects, we conducted a sensitivity analysis using the E-value method. The results indicated that an unmeasured confounder would need to increase the risk of death by 2.66-, 5.33-, or 2.06-fold to fully explain the observed relative risks in the “Surg−, Rad+,” “Surg+,Rad−,” and “Surg+,Rad+” groups, respectively.

To further clarify the finding that controlling for treatment reduced racial disparities in survival, we conducted two additional analyses. First, we modified Model 1 and Model 2 by removing the variable Metro (i.e., replacing Metro.rac with Race) and performed survival analyses. The results showed that the 95% confidence interval (CI) of the hazard ratio for Black versus White patients was 1.30–1.36 when treatment was not included and 1.21–1.26 when treatment was controlled for (Supplementary Figures S1 and S2). Second, using Gleason pattern information available for patients (N = 313,297) who had been recorded in the SEER database by 2010, we subdivided high-grade cancers into H− and H+ groups according to Gleason patterns (3 + 4 vs. others). Survival analyses using Model 1 and Model 2 showed that the estimated Rural–Urban Continuum Code-specific racial effects on survival (RR-1 values) were one third smaller, approximately, than that shown in Figure 1 and Figure 2, but the disparity pattern and the reduction in risk after controlling for treatment were nearly identical to those reported above (Supplementary Figures S3 and S4).

**Figure 3:**
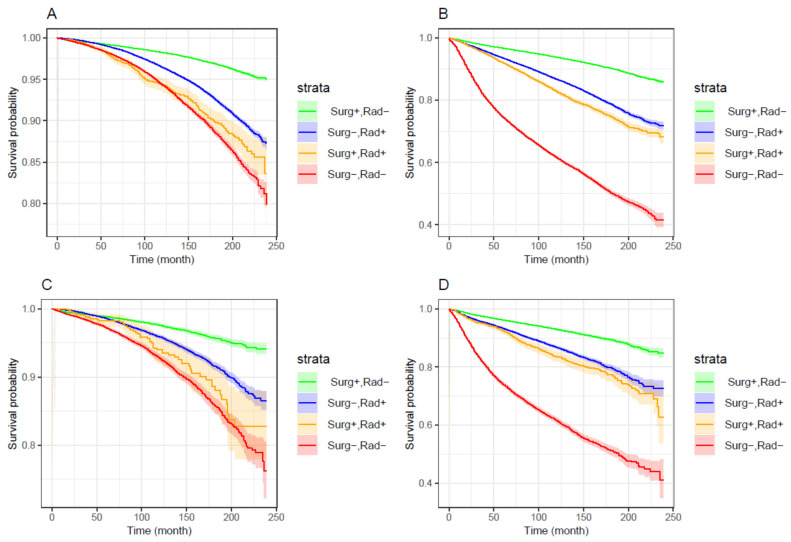
Kaplan–Meier (KM) analysis of prostate-specific mortality (or survival) for the groups defined by treatments. (**A**) KM curves for White patients with localized L grade prostate cancer. (**B**) KM curves for White patients with H grade or non-localized (regional and distant) prostate cancer. (**C**) KM curves for Black patients with localized L grade prostate cancer. (**D**) KM curves for Black patients with H grade or non-localized prostate cancer.

Figure 1 and Figure 2 showed the same advantage of order of treatments in improving survival. That is, the relative mortality risk increased in the order of surgery alone < radiotherapy alone < surgery-plus-radiotherapy < nonradical treatment. We further modeled the impact of treatments on PCSM with Kaplan–Meier survival curves. Based on the hazard ratios shown in Figure 1 and Figure 2, patients were temporally partitioned into two caner classes with different mortality risks: “H grade or non-localized (regional and distant)” and “localized L grade”. Figure 3 A–D demonstrated that, regardless races and the cancer classes, a common stratification pattern was that the curve of the “Surg+,Rad−” group was best, followed by the curves of the “Surg−,Rad+” and “Surg+,Rad+” groups, and then the curve of the “Surg−,Rad−” group at the last. For a specific race, the curve of “Surg+,Rad+” was close to the curve of “Surg−,Rad−” for patients with localized L grade cancer and was close to the curve of “Surg−,Rad+” for patients with H grade or non-localized cancer. In any scenario, the 95% confidence region of the curve of “Surg+,Rad−” was very narrow and did not overlap with the regions of the curves of other treatments.

### 3.2. Relationship Between Geographical Variation in PCSMR and Variation in Treatment

Data cells were defined by the combinations of Geographical areas (GARs; see Section 2), races, and the cancer classes used in generating Figure 3. Five-year Kaplan–Meier curve-based PCSMRs (death rate), average age at diagnosis (diagnosis age) and the proportion (surgery rate) of the patients who underwent surgery alone (without radiotherapy) were calculated for each data cell. With these summary metrics, a working dataset was established to infer the relationship between geographical variation in PCSMR and geographical variation in treatment. Regarding a specific cancer class, the information of GARs (as measurement units) that contained ≥100 Black and ≥100 White patients was used in the statistical analysis. 

**Figure 4:**
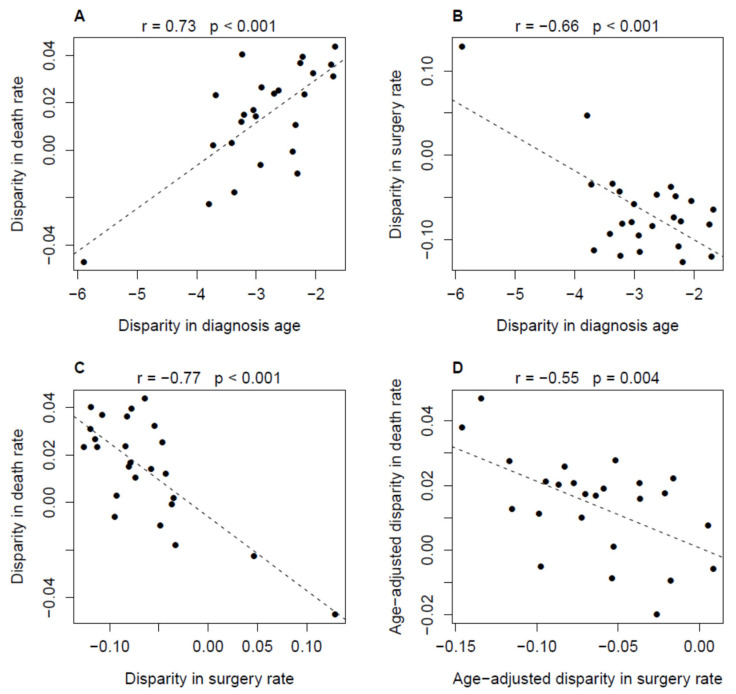
Association between disparity in death rate and disparity in treatment rate (surgery rate) for patients with non-localized H grade prostate cancer. (**A**,**B**): the association between disparity in age and disparity in death rate or surgery rate. (**C**): the association between disparity in surgery rate and disparity in death rate. (**D**): the association between disparity in age-adjusted surgery rate and age-adjusted disparity in death rate. Disparity in death rate is calculated as the difference in five-year PCSMR between Black and White subsets. Disparity in surgery rate is calculated as the difference in the fraction of PCa patients who underwent surgery alone, i.e., surgery but not pre- or post-operative radiotherapy, between the Black and White subsets. Each of 25 data points (dots) represents a geographical area (GAR). In each subfigure, the dash line is the regression line of the y-axis variable on the x-axis variable.

**Figure 5:**
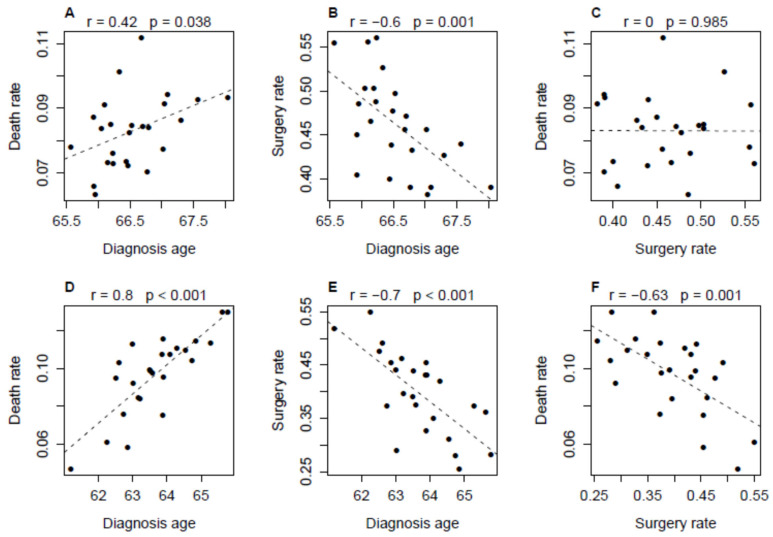
Correlations among geographical area-specific death rates, treatment rates (surgery rates) and average ages at cancer diagnosis for White patients (**A**–**C**) and Black patients (**D**–**F**) with the H grade or non-localized prostate cancer. Death rate is equivalent to five-rear PCSMR. Surgery rate is the fraction of patients who underwent surgery alone, i.e., surgery but not pre- or post-operative radiotherapy. Each of the 25 data points (dots) represents a geographical area (GAR). In each subfigure, the dash line is the regression line of the y-axis variable on the x-axis variable.

The plots in Figure 4 and Figure 5 show the results for patients with H grade or non-localized cancer. Disparity (Black patients’ value minus White patients’ value) in death rate and surgery rate were associated with disparity in diagnosis age (r = 0.73 or −0.66, *p* < 0.001; Figure 4A,B). There was a significant negative correlation between disparity in death rate and disparity in surgery rate (r = −0.77, *p* < 0.001; Figure 4C). The partial correlation was still significant (r = −0.55, *p* = 0.004) after the age factor was adjusted (using a regression model with age disparity as the predictor) for both death rate and surgery rate disparities. For both White and Black populations, there were a moderate (r = 0.42, *p* = 0.038; Figure 5A) or strong (r = 0.8, *p* < 0.01; Figure 5D) positive correlation between diagnosis age and death rate and a consistent strong negative correlation (r ≤ −0.6, *p* ≤ 0.001; Figure 5B,E) between diagnosis age and surgery rate. A significant negative correlation between surgery rate and death rate was demonstrated in Black patients (r = −0.63, *p* = 0.001, Figure 5F) but not in White patients (Figure 5C). This suggests that the association between the disparities in death rate and surgery rate was mainly due to the dependence of the variation in death rate on the variation in surgery rate in the Black population.

Except for the positive correlation between death rate and diagnosis age, the aforementioned relationships were not observed in patients with localized L grade cancer (Figures S5 and S6).

The following points are worth noting. First, the distribution pattern of data points (dots) in Figure 4C implied that, given the same surgery rate, the death rate of Black patients could be even lower than that of White patients. A mechanism underlying this observation was that, on average, Black patients were 1.5–6 years younger than White patients across the 25 GARs. Second, the relationship between death disparity and surgery disparity depicted in Figure 4 was generally held when an alternative x (ranging from 3 to 9)-year PCSMR was used as the death rate in the correlation analysis (Figures S7 and S8). Third, although large metropolitan areas such as New Orleans and Atlanta may contain substantial variation in treatment patterns and outcomes, our additional analysis showed that removing individual large metropolitan area from the aggregated dataset had only a minimal impact on the results of the correlation analyses.

Given the significant correlation between treatment disparity (X) and age disparity (Y) and their shared association with mortality disparity (Z), we examined whether X (or Y) mediated the effect of Y (or X) on Z. Regression-based mediation analyses were conducted using the mediate() function in the R package “mediation”. As shown in Supplementary Text S1, the direct effects of both X and Y on Z were significant (*p* = 0.05), whereas the assumed mediating effect of X (or Y) was not significant (*p* > 0.05). These results suggest that X and Y independently contribute to explaining Z, and that neither variable could explain away the effect of the other on mortality disparity.

### 3.3. Effects of Family History of Prostate Cancer (pros_fh) and Hormone Therapy

**Figure 6:**
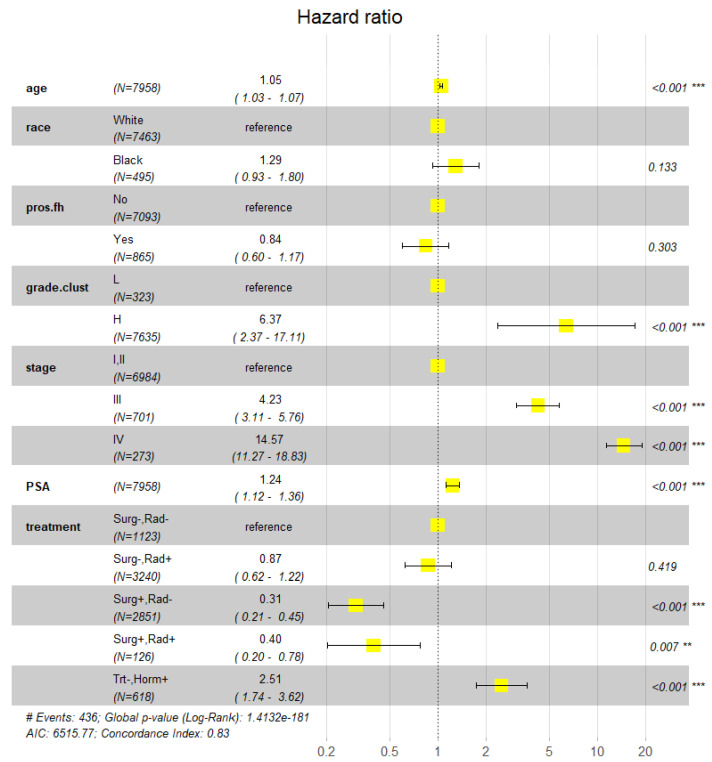
Effects of family history of prostate cancer (pros_fh) and hormone therapy on prostate cancer-specific mortality (survival). Explanatory variables in the model include age, race, pros_fh (binary indicator of prostate cancer family history), grade.clust (grade cluster), stage, PSA, and treatment. For each explanatory variable or categorical factor, the center of the filled yellow box represents the point estimate of the Hazard Ratio (HR) and the black “**|─|**” shape represents the 95% CI of the HR estimate. The ** and *** indicate *p* < 0.01 and 0.001, respectively.

This subsection reports the results from the analysis of the PLCO dataset using Model-3. Alongside treatment and other predictors for survival, pros_fh was included in the model. The reason was that we wondered if PCa family history was related to a special life environment or lifestyle that could drive or impede cancer progression. As shown in Figure 6, the effect of pros_fh on PCSM was not significant (RR 95% CI = 0.60–1.17, *p* = 0.303). A similar result (not presented here) was obtained when pros_fh was substituted with pros_fh_cn, which stood for the number of first-degree relatives with PCa. Among the five treatment groups, “Surg+,Rad−” had the best survival profile (RR 95% CI = 0.21–0.45, *p* < 0.001), largely consistent with the finding from the SEER dataset. However, the profile (RR 95% CI = 0.20–0.78, *p* < 0.007) of the “Surg+,Rad+” group was better than the profile (RR 95% CI = 0.62–1.22, *p* = 0.419) of the “Surg-,Rad+” group, representing a deviation from the results of the SEER dataset. Dropping PSA from the model had a minor impact on the last result. The analysis also showed that the mortality risk of the men in the “trt−,horm+” group, i.e., those who experienced hormone drug treatment but did not undergo surgery and/or radiotherapy, was greater than that of the men in the other four treatment groups (RR 95% CI = 1.74–3.62, *p* < 0.001). Such a result could not be inferred from the SEER dataset because of the lack of required information.

## 4. Discussion

Regional variations in cancer mortality have been widely reported in the past two decades [28,29,30]. Recently, we found that the racial disparities in PCa-specific survival and mortality rates in the United States varied geographically [11]. In the current study, we investigated the impact of treatments on PCSM, PCSMR and the relevant racial disparities. The primary results were that the relative risk in PCa mortality in Black patients (compared to Whites) estimated by a Cox PH regression model including treatment as an explanatory variable was obviously lower than that estimated by the reduced model excluding the variable; and, across geographical areas (GARs), the differences death rates between Black and White patients in PCSMR in patients with H grade or non-localized cancer were negatively correlated with the differences in the proportion of patients who underwent surgery alone (without radiotherapy). The correlation remained significant after adjusting for racial differences in age at diagnosis. Regression-based mediation analysis indicated that treatment disparity had a significant direct effect on mortality disparity but did not mediate the effect of age disparity. These findings support the hypothesis that geographical variation in treatment disparity may partially explain the variation in mortality disparity.

Regarding the impact of treatments on patient outcome, our analysis of the SEER data demonstrated that the relative mortality risk increased in the order of surgery alone < radiotherapy alone < surgery plus radiotherapy < nonradical treatment. While the ordering of surgery alone, radiotherapy alone and nonradical treatment were consistent with most of the previous studies on this topic, including those studies on the earlier versions of SEER data [31,32,33,34], the poor survival of patients who underwent both surgery and radiotherapy, especially in localized L grade cancer, was an interesting and somewhat unexpected result [35,36]. Moreover, it was not confirmed by the result of the PLCO data analysis, which showed that surgery plus (curative) radiotherapy was better than radiotherapy alone and somewhat poorer than surgery alone. At present, we could not dissect this puzzle due to the lack of required case-specific medical history information in the SEER database. We wondered whether the treatments of some patients in the “Surg+,Rad+” group were much delayed such that cancer had been metastasized, or they had experienced a treatment failure before undergoing the second treatment procedure. We also considered whether some patients in the SEER data received palliative rather than curative radiotherapy. However, the dataset lacks a variable to determine treatment intent. Based on the premise that palliative radiotherapy is typically administered to patients with advanced or metastatic cancer [37], we excluded individuals with cancer stage classified as “Distant” and performed survival analysis using Model 2. This modification had only a minor impact on the estimated treatment effects and did not alter the relative rankings. On the other hand, the puzzle may suggest something similar to the understanding that offering immediate adjuvant radiotherapy to all men with high-risk pathological factors could lead to overtreatment of those who would anyway be cancer-free and exposing them to unnecessary toxicity [38]. Anyway, the SEER data analysis raised an important question, that is, whether many patients who had L grade localized cancer and underwent surgery-plus-radiotherapy were overtreated in the United States? The doubt could hardly be alleviated by the relevant result obtained from the PLCO dataset because the set was predominated by men with H grade cancer, and the hazard ratio of “Surg+,Rad+” treatment had a quite wide 95% CI (0.20–0.78) due to the small group size (N = 126). In summary, the advantage of radiotherapy alone and surgery plus radiotherapy in preventing mortality remains uncertain.

Previous studies showed that neoadjuvant hormone therapy prior to prostatectomy did not improve the overall survival of PCa patients, but hormone therapy combined with either prostatectomy or radiotherapy is associated with significant clinical benefits in patients with local or locally advanced PCa [39,40]. Our analysis of the PLCO dataset demonstrated that the patients who were offered hormone therapy but did not undergo surgery or radiotherapy had very poor survival. The relative risk of mortality in these men was even higher than that in the nonradical treatment group. While the medical contexts and health statuses of the hormone therapy cases, which may be related to the elevated mortality, could not be inferred from the available data, cancer grade and stage should not be the major contributing factors to the RR estimate because it was included in the statistical model. Nevertheless, the undesirable outcomes of hormone therapy could help explain the high PCSMR in Black men. In an additional (unpublished) study using a dataset extracted from database of Ochsner Health (https://www.ochsner.org/), Louisiana, we found that Black patients with malignant prostate cancer were more frequently offered bicalutamide and/or leuprolide (two most widely used hormone anticancer drugs in PCa patients) treatment than White patients, compatible with the high mortality in the Black population.

Aging and PCa family history are two leading contributing factors to men’s susceptibility to cancer [41,42,43]. The analysis of the SEER dataset showed that racial disparity in average age within a geographical area was associated with the disparity in PCSMR and that adjusting for age alleviated the correlation between mortality disparities and treatment disparities. This indicates that the influence of age on PCSMR disparity was partially mediated by treatment disparity. The analysis of the PLCO dataset demonstrated that PCSM and the effects of treatments on PCSM were independent of PCa family history, which is commonly determined by genetic and familiar environmental factors. However, this does not mean that there is no genetic component in the survival of PCa patients. Previous studies and our recent work identified a few dozen genetic variants that were significantly or moderately associated with PCSM but not with PCa incidence [44,45,46]. In contrast, none of the ~270 documented PCa risk SNP variants have been associated with PCSM [47,48]. These results suggest that the two traits have different genetic architectures. Similarly, the potential familiar environmental factors for PCSM may deviate from those for PCa susceptibility.

Economic disadvantage is usually considered a major contributing component of racial disparity in (prostate) cancer mortality [49]. The analysis of the SEER dataset showed that while the survival of PCa patients in counties with low median annual household income was poorer than that of those in counties with high income, the effect of the income variable on PCSM was much smaller than the effects of the variables representing cancer grade, stage and treatment, even when the relative risk was estimated using the reduced model excluding the treatment variable. This implies that the influence of socioeconomic factors only exerted a limited impact on the accession, choice, and/or adoption of treatment procedures.

There were a few major limitations in this study. First, because a variable representing the county or parish where a patient lived was not available in the version of the SEER database used in this study (and in other currently available versions of the data), we could not follow the manner in reference [19] to define more specific county-level regions as experimental or measurement units in calculating mortality rates and treatment rates. Due to this limitation, the size of the aggregated dataset was relatively small, and the differences among communities might be compromised by the heterogeneity within a large Rural–Urban Continuum such as New Orleans, which could lead to a reduction in statistical power. Accordingly, there was a possibility that we failed to reveal the potential correlation between geographical area-specific disparity in death rate and the corresponding disparity in treatment rate in localized low-grade PCa. Second, given the rapid rise in patients managed on active surveillance in the United States over the last 10–15 years, our finding that the patients who did not undergo radical treatment had poorest survival, regardless of race and cancer aggressive classes defined by grade and stage, may be affected when more updated data is used. Last, patients receiving different treatments may differ in important, unmeasured ways, which could bias our estimates of treatment effects on survival and the relationship between treatment disparity and mortality disparity. For example, unmeasured confounders such as diabetes could introduce bias, as diabetes may limit a patient’s ability to receive or complete curative treatment, increase PCa-specific mortality, and varies in prevalence across racial groups [50,51]. 

## 5. Conclusions

This study investigated the impact of treatments on PCa-specific mortality and the relevant disparities. The primary findings were as follows: the relative mortality risk (RR) of patients undergoing surgery alone was significantly lower than that of patients receiving radiotherapy alone or a combination of surgery and radiotherapy; Black patients’ RR estimated by the model that included an explanatory variable for treatments was substantially smaller than that estimated by the reduced model excluding the variable; across geographical areas, racial disparities in mortality were associated with disparities in treatment; and treatment disparity had a significant direct effect on mortality disparity and did not mediate the effect of age disparity. These findings shed light on the etiology of the poorer survival of Black PCa patients. In particular, they provide supportive evidence for the hypothesis that geographical variation in treatment disparity may partially explain the variation in mortality disparity. However, due to the potential presence of unmeasured confounders, the causal role of treatment disparity in driving mortality disparity remains uncertain.

## Figures and Tables

**Table 1:** Summary of samples relevant to the considered patient traits, cancer characteristics and community attributes in the SEER and PLCO datasets.

	SEER		PLCO	
	White	Black	White	Black
Age at diagnosis				
35–55	72,123	20,957	397	32
56–80	535,347	87,230	7066	463
>80	29,623	2652	0	0
Grade cluster				
H (Gleason score ≥ 7)	255,587	47,451	7160	475
L (Gleason score < 7)	381,506	63,388	303	20
Stage				
Localized in SEER data; I, II in PLCO data	529,315	93,483	6559	425
Regional in SEER data; III in PLCO data	87,134	12,494	666	35
Distant in SEER data; IV in PLCO data	20,644	4862	238	35
Rural–Urban Continuum Code				
M1 = Counties in metropolitan areas ge 1 million pop	361,984	70,358		
M2 = Counties in metropolitan areas of 0.25–1 million pop	133,085	20,603		
M3 = Counties in metropolitan areas of lt 250 thousand pop	58,904	9500		
M4 = Nonmetropolitan counties	83,120	10,378		
Treatment (therapy)				
Surg−,Rad−	148,822	30,398	1061	62
Surg−,Rad+	210,414	41,218	3000	240
Surg+,Rad−	257,761	36,000	2708	143
Surg+,Rad+	20,096	3223	119	7
Surg−,Rad−,Horm+)	----	----	575	43
Income (County median household income, inflation adj to 2019)				
INC.L < $50,000	77,359	24,277		
INC.M = $50,000−69,999	286,861	54,016		
INC.H ≥ $70,000	272,873	32,546		
Prostate family history				
No			6641	452
Yes			763	39
Number of first-degree relatives with prostate cancer ≥ 2			59	4
PSA, closest to diagnosis				
<5			2259	115
≥5 & <10			3370	220
≥10 & <40			1210	109
≥40			163	27
Missing			458	24

## Data Availability

The datasets used are deposited in the SEER database (https://seer.cancer.gov/, accessed on 19 September 2022) and the PLCO database (https://cdas.cancer.gov/plco/, accessed on 24 August 2019). Access to these data is controlled by the corresponding NIH data access committees. The corresponding authors of this study obtained approval from the committees to access and analyze these data.

## Supplementary Materials

The following supporting information can be downloaded at https://www.mdpi.com/article/10.3390/cancers18060983/s1. Figure S1: Analysis of racial disparities in prostate cancer-specific mortality (or survival) using a modified version (i.e., replacing Metro.rac with Race) of Model-1, which does not include an explanatory variable for treatments; Figure S2: Analysis of racial disparities in prostate cancer-specific mortality (or survival) using a modified version (i.e., replacing Metro.rac with Race) of Model-2, which includes an explanatory variable for treatments; Figure S3: Analysis of racial disparities in prostate cancer-specific mortality (or survival) using a modified version (i.e., subdividing high-grade cancers into H− and H+ groups according to Gleason patterns (3+4 vs. others)) of Model-1, which does not include an explanatory variable for treatments; Figure S4: Analysis of racial disparities in prostate cancer-specific mortality (or survival) using a modified version (i.e., subdividing high-grade cancers into H− and H+ groups according to Gleason patterns (3+4 vs. others)) of Model-2, which includes an explanatory variable for treatments; Figure S5: Association between disparity in death rate and disparity in treatment rate (surgery rate) for patients with localized L grade prostate cancer; Figure S6: Correlations among geographical area-specific death rates, treatment rates (surgery rates) and average ages at cancer diagnosis for White patients (A–C) and Black patients (D–F) with localized L grade prostate cancer; Figure S7: Association between disparity in death rate (three-year PCSMR) and disparity in treatment rate (surgery rate) for patients with non-localized or H grade prostate cancer; Figure S8: Association between disparity in death rate (nine-year PCSMR) and disparity in treatment rate for patients with non-localized or H grade prostate cancer; Text S1: Results of mediation analyses.
