# Supplementary material for: Modeling the Effect of Treatments on Prostate Cancer-Specific Mortality and the Relevant Geographical Variation and Racial Disparities
Source: Cancers (Basel). 2026 Mar 18;18(6):983. doi: 10.3390/cancers18060983 (PMC13024551; doi:10.3390/cancers18060983)
Supplement: Supplementary file 1 [file cancers-18-00983-s001.zip › cancers-4142440-Supplementary.pdf]

Supplementary information for "Modeling the effect of treatment on the prostate cancer-specific mortality and the relevant racial disparities"

Wensheng Zhang[1.2], Christopher Williams[3], Guangdi Wang[4], Kun Zhang[1,2]

[1]Bioinformatics Core of Xavier RCMI Center of Cancer Research, Xavier University of Louisiana, New Orleans, LA 70125, USA

[2] Department of Computer Science, Xavier University of Louisiana, New Orleans, LA 70125, USA

[3] School of Pharmacy, Xavier University of Louisiana, New Orleans, LA 70125, USA

[4] Department of Chemistry, Xavier University of Louisiana, New Orleans, LA 70125, USA

Content:

Figure S1, page 2

Figure S2, page 3

Figure S3, page 4

Figure S4, page 5

Figure S5, page 6

Figure S6, page7

Figure S7, page 8

Figure S8, page 9

Text S1, page 10

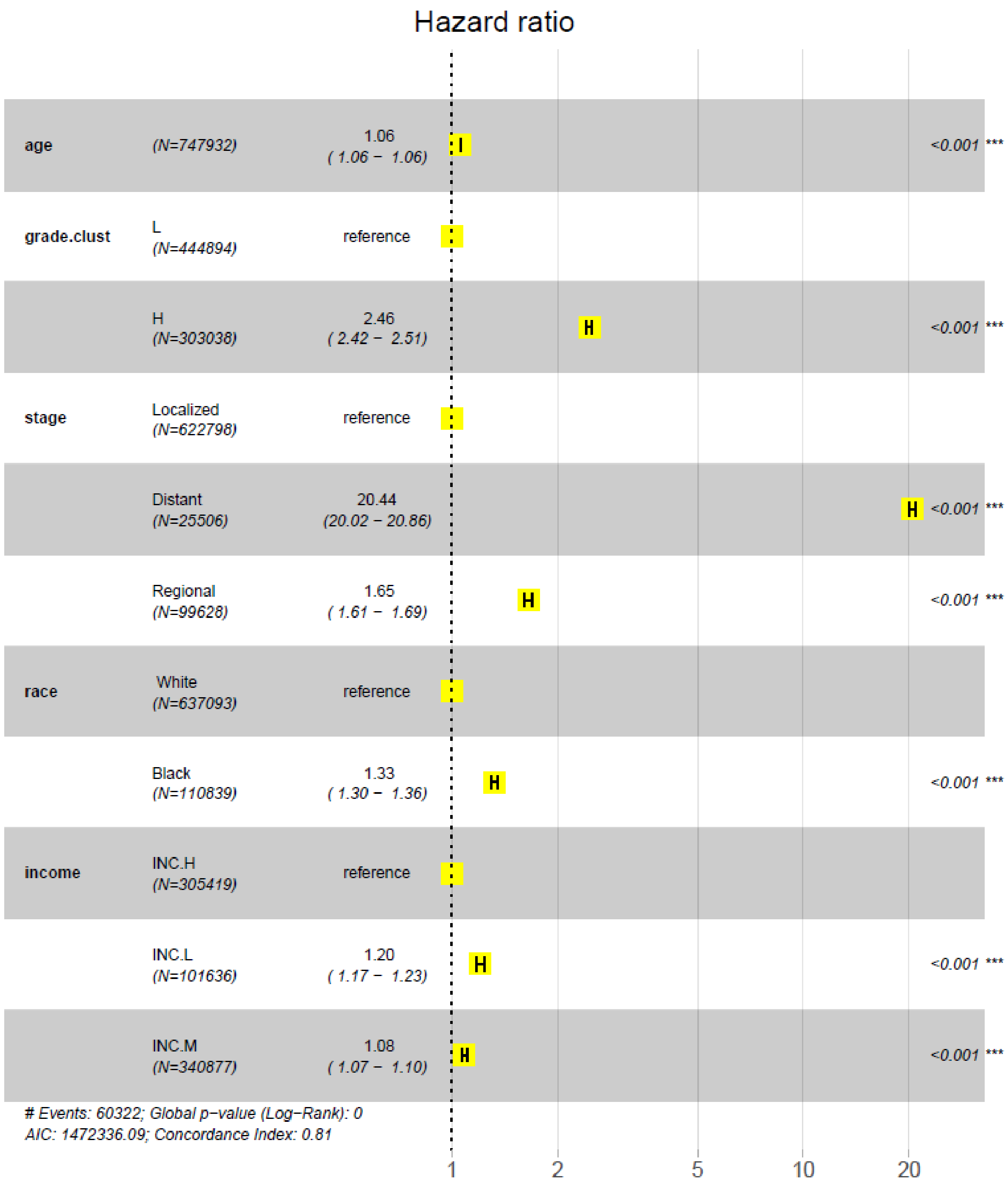

Figure S1 Analysis of racial disparities in prostate cancer-specific mortality (or survival) using a modified version (i.e., replacing Metro.Rac with Race) of Model-1, which does not include an explanatory variable for treatments. The explanatory variables in the model include age, grade.clust (grade cluster), stage, **race**, and (county-level median annual household) income. For each explanatory variable or categorical factor, the center of the filled yellow box represents the point estimate of the Hazard Ratio (HR) and the black "|—|" shape represents the 95% CI of the HR estimate. The *** indicates p < 0.001.

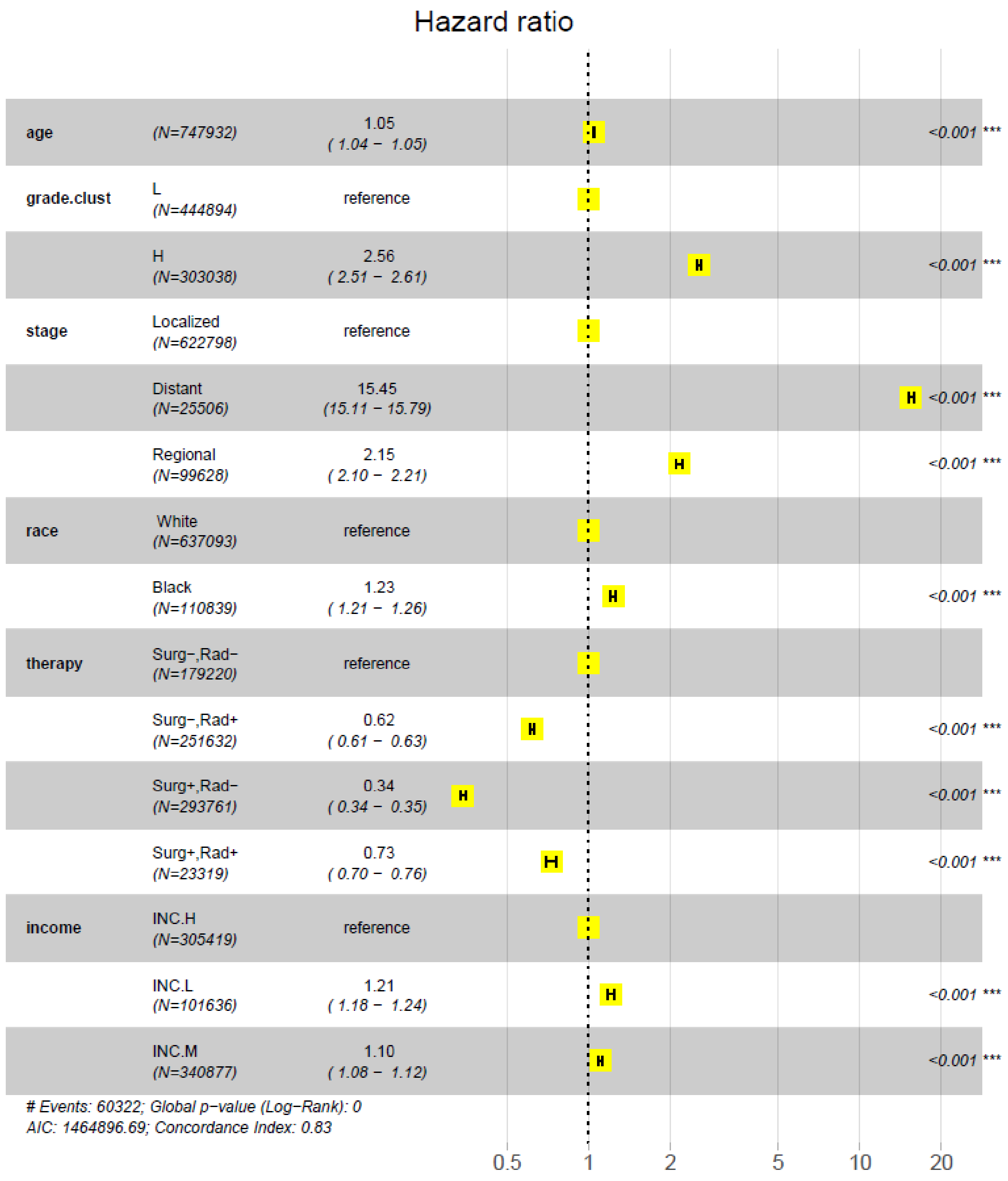

Figure S2 Analysis of racial disparities in prostate cancer-specific mortality (or survival) using a modified version (i.e., replacing Metro.rac with Race) of Model-2, which includes an explanatory variable for treatments. Besides treatment (therapy), the explanatory variables in the model include age, grade.clust (grade cluster), stage, **race,** and (county-level median annual household) income. For each explanatory variable or categorical factor, the center of the filled yellow box represents the point estimate of the Hazard Ratio (HR) and the black "|—|" shape represents the 95% CI of the HR estimate. The *** indicates $p < 0.001$.

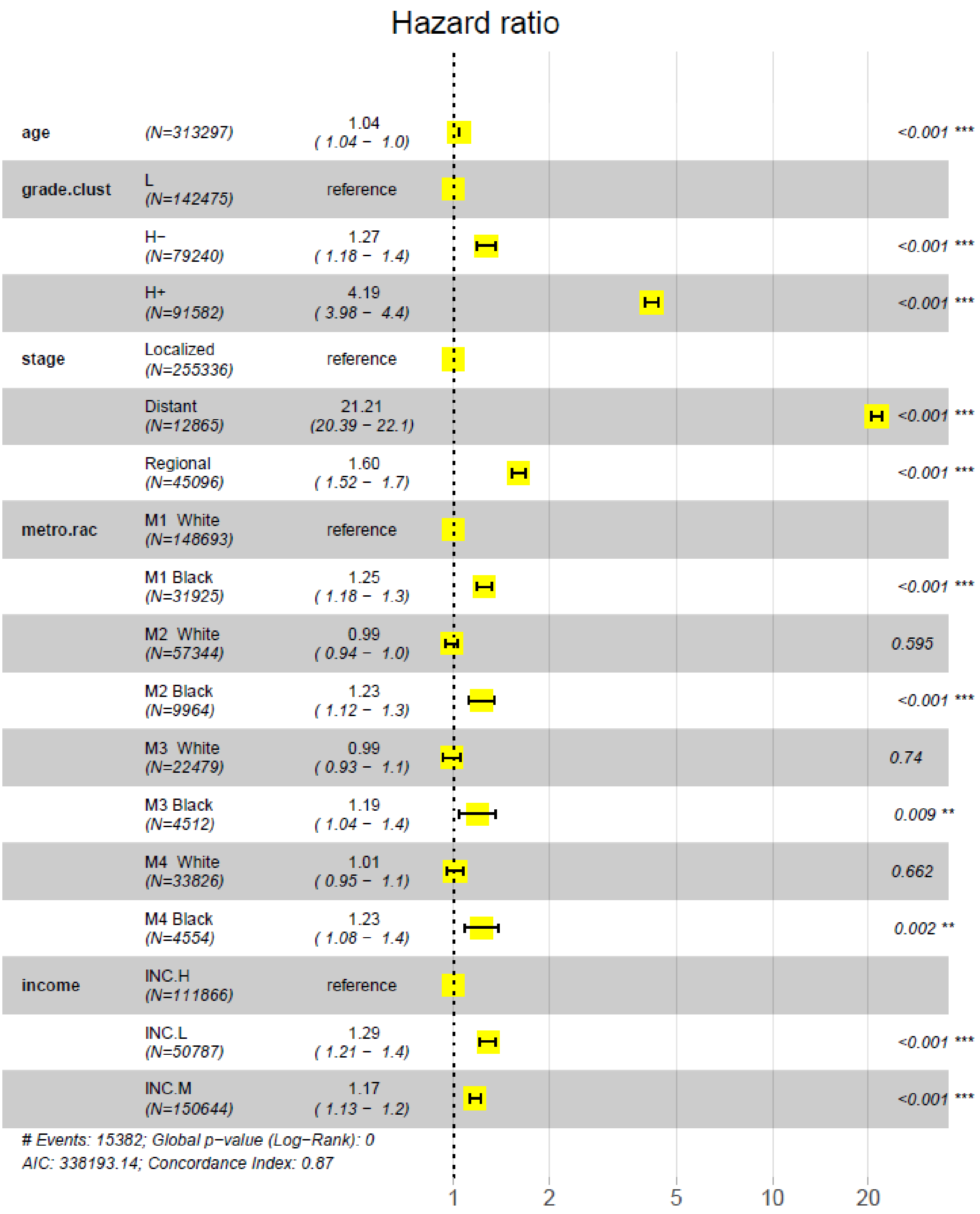

Figure S3 Analysis of racial disparities in prostate cancer-specific mortality (or survival) using a modified version (i.e. subdividing high-grade cancers into H− and H+ groups according to Gleason patterns (3+4 vs. others)) of Model-1, which does not include an explanatory variable for treatments. The explanatory variables in the model include age, grade.clust (grade cluster), stage, metro.rac, and (county-level median annual household) income. For each explanatory variable or categorical factor, the center of the filled yellow box represents the point estimate of the Hazard Ratio (HR) and the black "|−|" shape represents the 95% CI of the HR estimate. The ** and *** indicate $p < 0.01$ and 0.001, respectively.

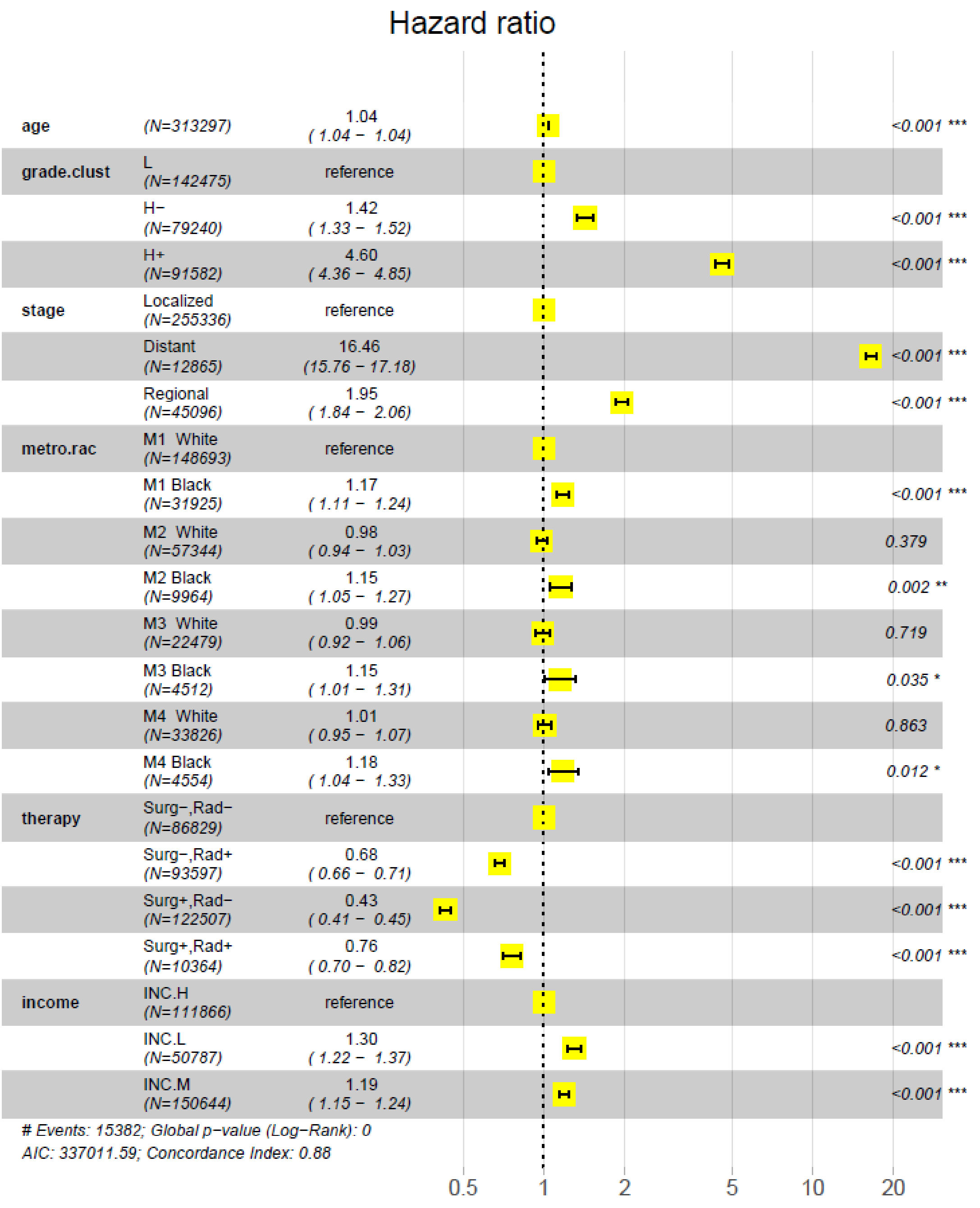

Figure S4 Analysis of racial disparities in prostate cancer-specific mortality (or survival) using a modified version (i.e. subdividing high-grade cancers into H− and H+ groups according to Gleason patterns (3+4 vs. others)) Model-2, which includes an explanatory variable for treatments. Besides treatment (therapy), the explanatory variables in the model include age, grade.clust (grade cluster), stage, metro-rac, and (county-level median annual household) income. For each explanatory variable or categorical factor, the center of the filled yellow box represents the point estimate of the Hazard Ratio (HR) and the black "|—|" shape represent the 95% CI of the HR estimate. The *, **, and *** indicate to $p < 0.05$, 0.01, and 0.001, respectively.

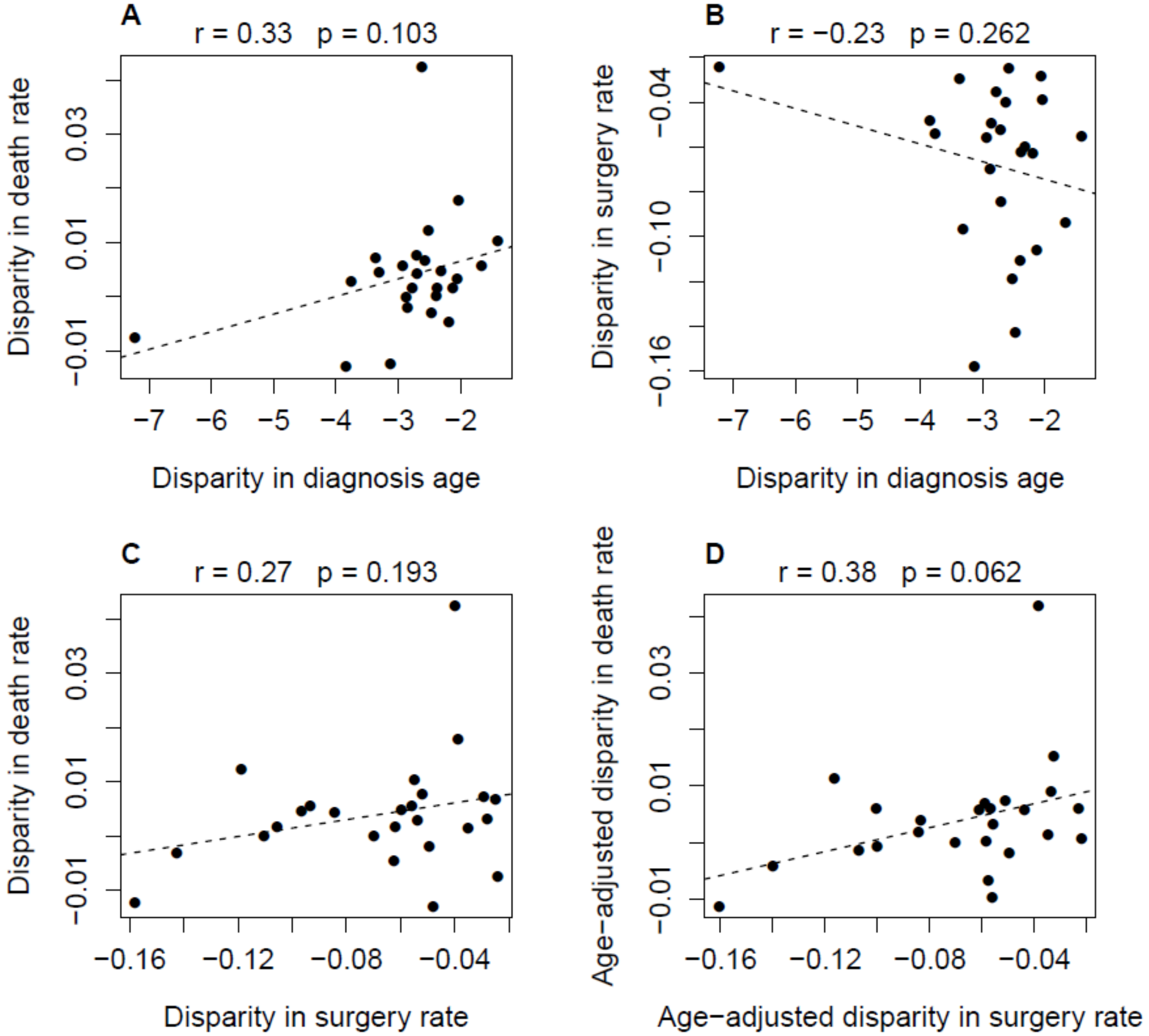

Figure S5 Association between disparity in death rate and disparity in treatment rate (surgery rate) for patients with localized L grade prostate cancer. (A, B): the association between disparity in age and disparity in death rate or surgery rate. (**C**): the association between disparity in surgery rate and disparity in death rate. (**D**): the association between disparity in age-adjusted surgery rate and age-adjusted disparity in death rate. Disparity in death rate is calculated as the difference in five-year PCSMR between Black and White subsets. Disparity in surgery rate is calculated as the difference in the fraction of PCa patients who underwent surgery alone, i.e. surgery but not pre- or post-operative radiotherapy, between the Black and White subsets. Each of the 25 data points (dots) represents a geographical area (GAR). In each subfigure, the dash line is the regression line of the y-axis variable on the x-axis variable.

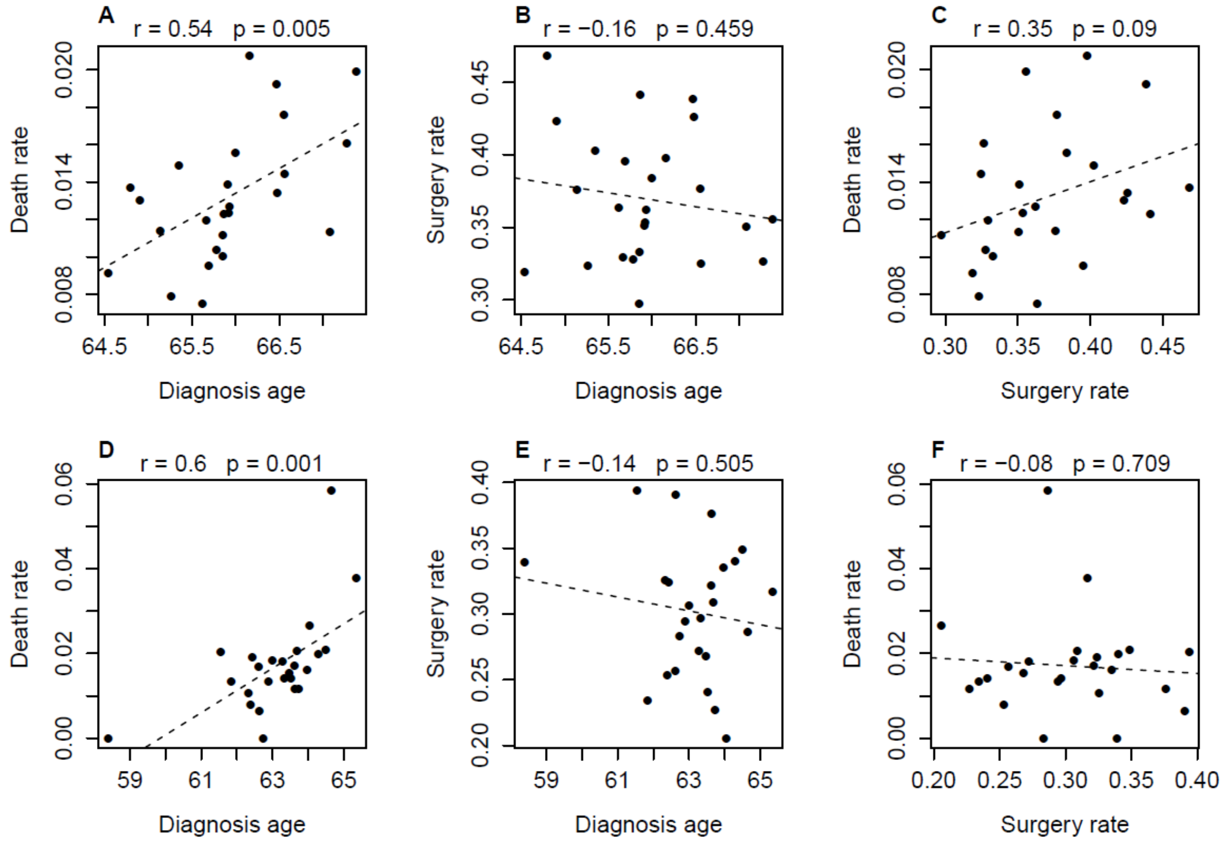

Figure S6 Correlations among geographical area-specific death rates, treatment rates (surgery rates) and average ages at cancer diagnosis for Whites (A, B, C) and Blacks (D, E, F) with localized L grade prostate cancer. Death rate is equivalent to five-rear PCSMR. Surgery rate is the fraction of patients who underwent surgery alone, i.e. surgery but not pre- or post-operative radiotherapy. Each of the 25 data points (dots) represents a geographical area (GAR). In each subfigure, the dash line is the regression line of the y-axis variable on the x-axis variable.

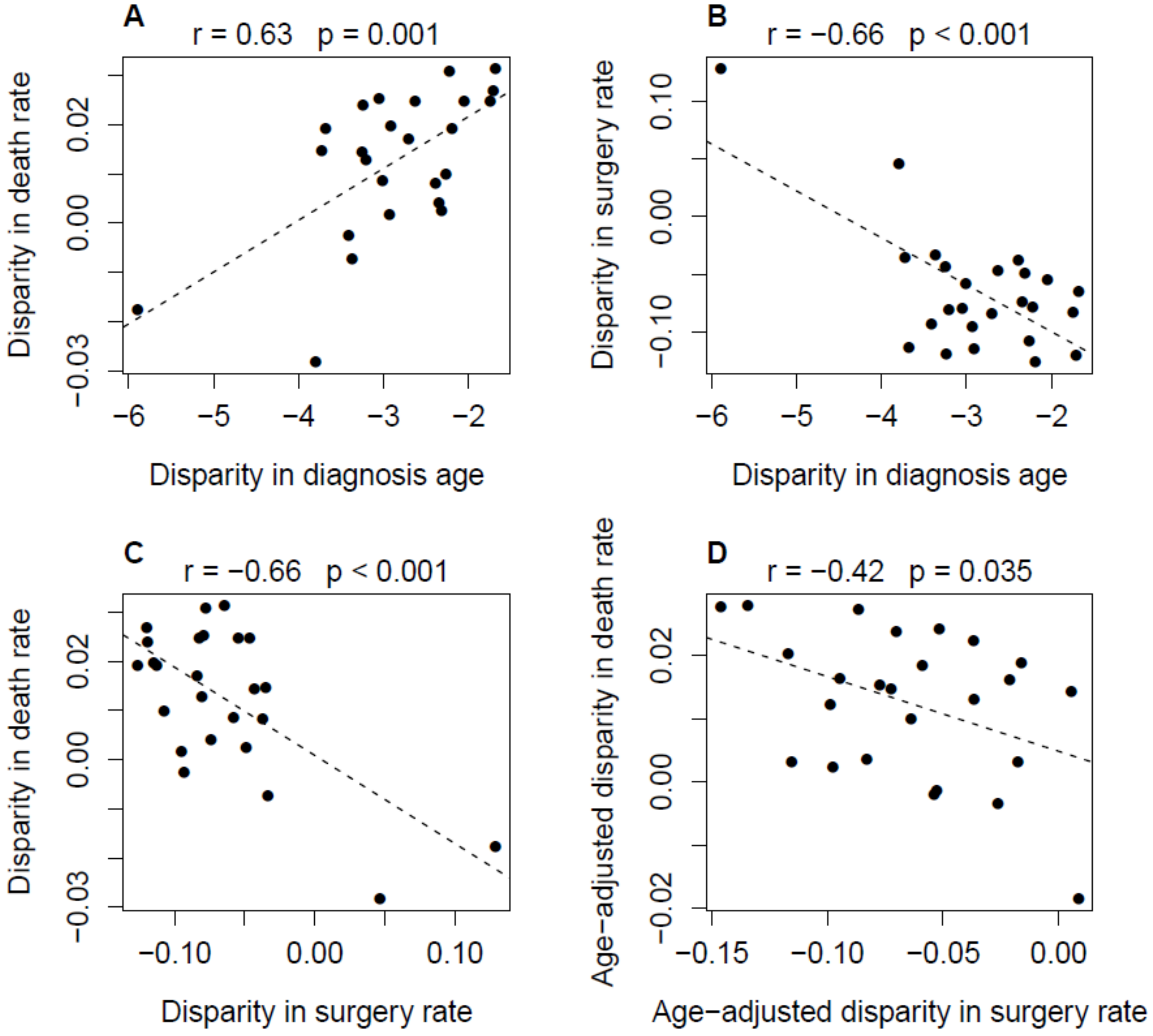

Figure S7 Association between disparity in death rate (three-year PCSMR) and disparity in treatment rate (surgery rate) for patients with non-localized or H grade prostate cancer. (**A, B**): the association between disparity in age and disparity in death rate or surgery rate. (**C**): the association between disparity in surgery rate and disparity in death rate. (**D**): the association between disparity in age-adjusted surgery rate and age-adjusted disparity in death rate. Disparity in death rate is calculated as the difference in three-year PCSMR between Black and White subsets. Disparity in surgery rate is calculated as the difference in the fraction of PCa patients who underwent surgery alone, i.e. surgery but not pre- or post-operative radiotherapy, between the Black and White subsets. Each data point (dot) represents a geographical area (GAR). In each subfigure, the dash line is the regression line of the y-axis variable on the x-axis variable.

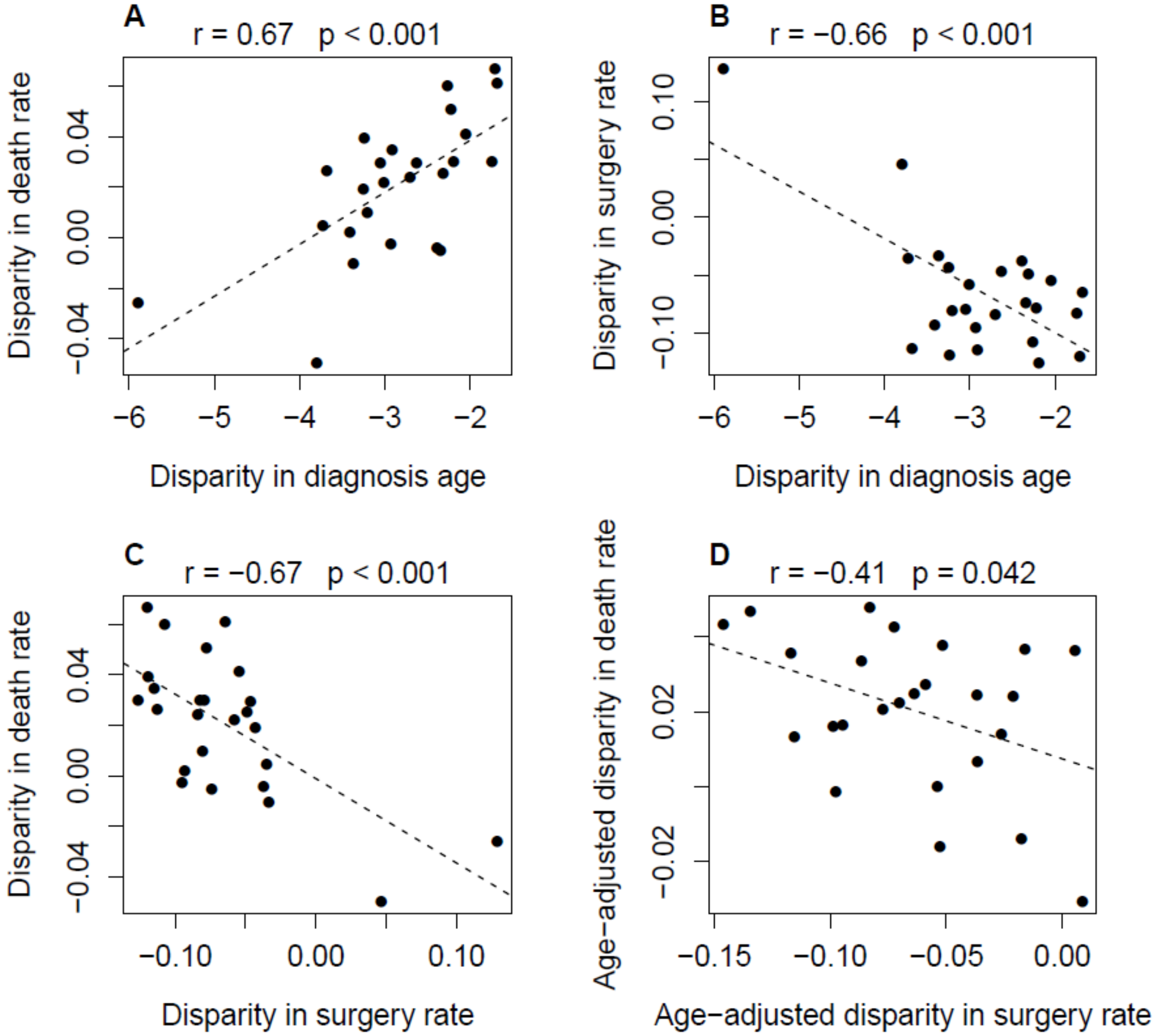

Figure S8 Association between disparity in death rate (nine-year PCSMR) and disparity in treatment rate (surgery rate) for patients with non-localized or H grade prostate cancer. (**A, B**): the association between disparity in age and disparity in death rate or surgery rate. (**C**): the association between disparity in surgery rate and disparity in death rate. (**D**): the association between disparity in age-adjusted surgery rate and age-adjusted disparity in death rate. Disparity in death rate is calculated as the difference in nine-year PCSMR between Black and White subsets. Disparity in surgery rate is calculated as the difference in the fraction of PCa patients who underwent surgery alone, i.e. surgery but not pre- or post-operative radiotherapy, between the Black and White subsets. Each data point (dot) represents a geographical area (GAR). In each subfigure, the dash line is the regression line of the y-axis variable on the x-axis variable.

Text S1 Results of mediation analyses

Results of mediation analysis-1

Nonparametric Bootstrap Confidence Intervals with the Percentile Method

Disparity in surgery rate as explanatory variable; disparity in diagnosis age as mediation variable; disparity in death rate as dependent variable.

Sample Size Used: 25; Simulations: 500

	Estimate	95% CI Lower	95% CI Lower	p-value
ACME	-0.104	-0.235	0.028	0.124
ADE	-.206	-0.357	-0.056	0.012
Total effect	-0.310	-0.379	-0.179	< 0.001
Prop. Mediated	0.336	-0.098	0.0.774	0.124

ACME: average causal mediation effect; ADE: Average direct effect; Prop. Mediated: proportion of mediated effect.

Results of mediation analysis-2

Nonparametric Bootstrap Confidence Intervals with the Percentile Method

Disparity in diagnosis age as explanatory variable; disparity in surgery rate as mediation variable; disparity in death rate as dependent variable.

Sample Size Used: 25; Simulations: 500

	Estimate	95% CI Lower	95% CI Lower	p-value
ACME	0.008	0.001	0.016	0.092
ADE	0.01	0.001	0.019	0.032
Total effect	0.018	0.01	0.023	0.004
Prop. Mediated	0.464	-0.086	0.937	0.072

ACME: average causal mediation effect; ADE: Average direct effect; Prop. Mediated: proportion of mediated effect.
